# Intron 3 Sixteen Base Pairs Duplication Polymorphism of *P53* Contributes to Breast Cancer Susceptibility: Evidence from Meta-Analysis

**DOI:** 10.1371/journal.pone.0061662

**Published:** 2013-04-19

**Authors:** Dongmei Wu, Zhizhong Zhang, Haiyan Chu, Ming Xu, Yao Xue, Haixia Zhu, Zhengdong Zhang

**Affiliations:** 1 Department of Environmental Genomics, Jiangsu Key Lab of Cancer Biomarkers, Prevention and Treatment, Cancer Center, Nanjing Medical University, Nanjing, China; 2 Department of Genetic Toxicology, the Key Laboratory of Modern Toxicology of Ministry of Education, School of Public Health, Nanjing Medical University, Nanjing, China; 3 Department of Neurology, Jinling Hospital, Nanjing University School of Medicine, Nanjing, China; 4 Core Laboratory, Nantong Cancer Hospital, Nantong, China; Northwestern University Feinberg School of Medicine, United States of America

## Abstract

**Background:**

*P53* is a tumor suppressor gene and plays important role in the etiology of breast cancer. Intron 3 sixteen-bp duplication polymorphism of *p53* has been reported to be associated with breast cancer risk. However, the reported results remain conflicting rather than conclusive.

**Methods:**

A meta-analysis including 19 case-control studies was performed to address this issue. Odds ratios (ORs) with 95% confidence intervals (CIs) were adopted to evaluate the association.

**Results:**

The overall results suggested that the variant genotypes were associated with a significantly increased breast cancer risk (Del/Ins vs Del/Del: OR = 1.18, 95% CI: 1.00–1.40; Ins/Ins vs Del/Del: OR = 1.42, 95% CI = 1.09–1.84; Ins/Ins+Del/Ins vs Del/Del: OR = 1.21, 95% CI = 1.03–1.41). When stratifying by sample size of studies, a significantly elevated risk was also observed among large sample studies (>500 subjects) but not among small sample studies (≤500 subjects).

**Conclusion:**

These results suggested that the 16-bp duplication polymorphism of *p*53 may contribute to susceptibility to breast cancer. Additional well-designed large studies were required to validate this association in different populations.

## Introduction

Breast cancer is the most commonly diagnosed cancer and a predominate cause of death in female population worldwide [1]. Although many risk factors for breast cancer have been identified, such as the genetic predisposition and estrogen level, the molecular mechanisms related to breast carcinogenesis remain under investigation [2], [3].

**Figure 1:**
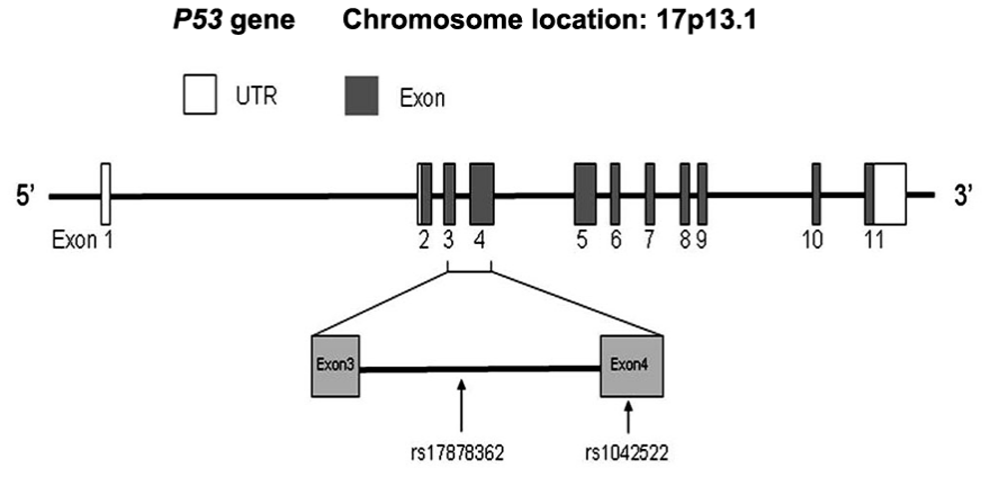
Gene structure of *P53* gene and location of intron 3 16-bp duplication polymorphism (rs17878362).

The *p53* tumor suppressor gene, designated as the guardian of the genome, is the most frequently mutated gene in different types of cancers [4]. It is reported that the *p53* gene is mutated in 20%–30% of the sporadic breast cancer [5]. These mutations can affect the functions of p53 protein as a transcription factor, and consequently many crucial functions such as DNA repair, cell cycle control, and apoptosis may be altered. Besides mutations, many single nucleotide polymorphisms (SNPs) have been identified in *p53* gene. The most informative one is the codon 72 Arg>Pro polymorphism (rs1042522), which results in structural alteration of the p53 protein and consequently affect its functions [6]. To date, the SNP rs1042522 has been found to be associated with risk of various cancers, including breast cancer, in different populations [7]–[9]. Moreover, the *p53* gene also contains several polymorphisms in non-coding region. Among these, the 16-bp duplication polymorphism (rs17878362) within intron 3 has been widely analyzed as possible cancer susceptibility modifiers (Figure 1). Gemignani *et al.* [10] found that the 16-bp Ins allele led to lower level of *p53* transcript, suggesting that this polymorphism causes an alteration in mRNA processing, which provides a possible molecular basis for the associated increased risk of developing cancer.

Over the last two decades, a number of case-control studies were conducted to investigate the association between the SNP rs17878362 and breast cancer risk. But these studies reported conflicting results. Since a single study might have been underpowered to detect the overall effects, a quantitative synthesis of the pooled data from different studies was deemed important to provide evidence for this association.

Thus, we carried out a meta-analysis on all eligible case-control studies to estimate the overall breast cancer risk of *p53* intron 3 duplication polymorphism as well as to quantify the between-study heterogeneity and potential bias.

## Materials and Methods

### Literature search and data extraction

To identify all studies that examined the association of *p53* intron 3 duplication polymorphism with breast cancer risk, we conducted a literature search of the PubMed and Embase database without a language limitation (the last search update was September 15, 2012, using the search terms “p53”, “polymorphism”, “variant”, and “breast cancer”). Review articles were hand-searched to find additional eligible studies and only published studies with full-text articles were included. Studies included in our meta-analysis have to meet the following criteria: (1) use a case-control design and (2) contain available genotype frequency. Major reasons for exclusion of studies were: (1) genotype frequency was not reported (2) duplicate of previous publication and (3) abstract, comment and review.

Information was carefully extracted from all eligible publications independently by two authors (D.W. and Z.Z.). For each included study, the following information was sought: the first author's last name, year of publication, country of origin, ethnicity, numbers of genotyped cases and controls, source of control groups (population- or hospital-based controls), genotyping methods, and quality control.

### Statistical analysis

For control group of each study, the allelic frequency was calculated and the observed genotype frequencies of the SNP rs17878362 were assessed for Hardy-Weinberg equilibrium using the χ^2^ test. The strength of the association between the SNP rs17878362 and breast cancer risk was measured by odds ratios (ORs) with 95% confidence intervals (CIs). We first estimated the risks of the variant Ins/Del and Ins/Ins genotypes on breast cancer, compared with the wild-type Del/Del homozygote, and then evaluated the risks of (Ins/Ins+Ins/Del) vs Del/Del and Ins/Ins vs (Ins/Del+Del/Del) on breast cancer, assuming dominant and recessive effects of the variant Ins allele, respectively. Stratified analyses were also performed by ethnicity, source of controls and sample size (≤500 and >500 subjects).

For the heterogeneity test, a fixed-effect model (the Mantel-Haenszel method) [11] was used when *P*>0.05, otherwise a random-effect model (the DerSimonian and Laird method) was used [12].

Cumulative meta-analysis were conducted to provide a framework for updating a genetic effect from all studies and to measure how much the genetic effect alters as evidence accumulates.

Sensitivity analyses were performed to assess the stability of the results, namely, a single study in the meta-analysis was deleted each time to reflect the influence of the individual data set to the pooled OR. Funnel plots and Egger's linear regression tests were used to provide diagnosis of the potential publication bias [13]. All analyses were done with Stata software (version 11.0; StataCorp LP, College Station, TX), using two-sided *P* values.

## Results

### Characteristics of studies

**Figure 2:**
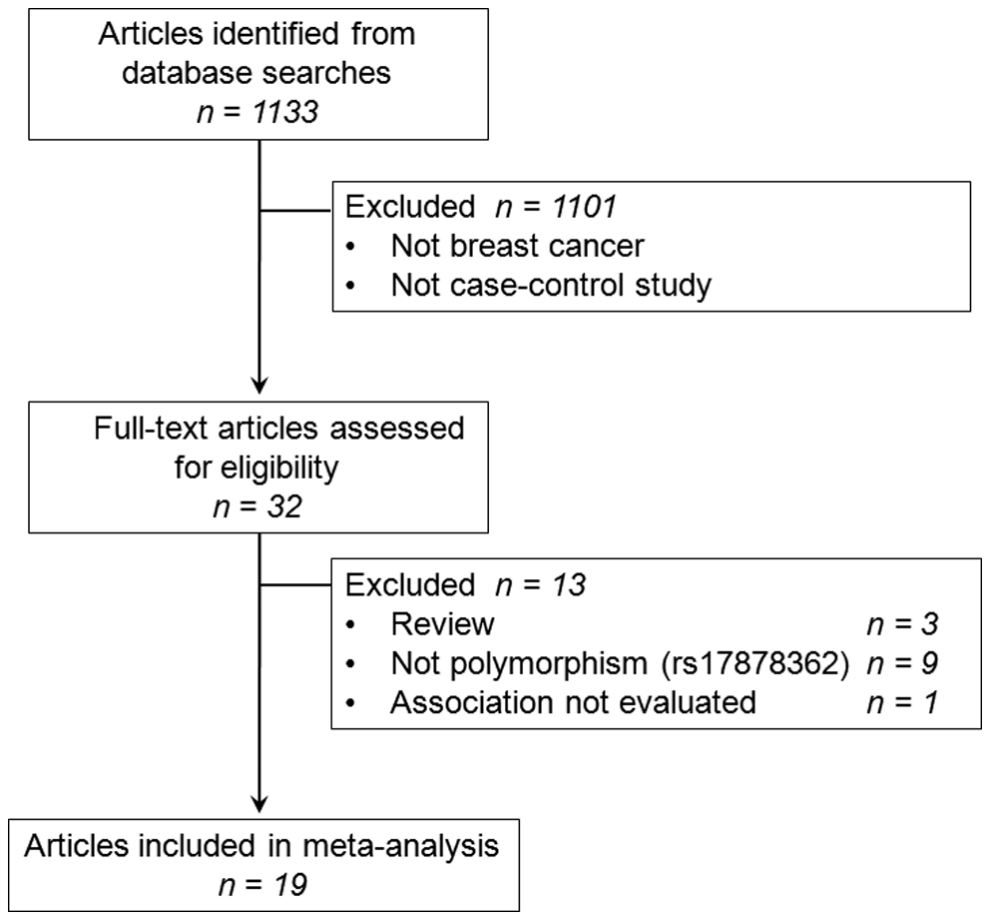
Flow diagram of the literature search.

The flow chart in Figure 2 summarizes the process of study selection. A total of 19 studies were retrieved based on the search criteria [14]–[32]. Study characteristics are summarized in Table 1. Among the 19 eligible case-control studies, there were 4479 breast cancer cases and 4683 controls. For race distribution, there were 6 studies of Europeans, 4 studies of Asians and 1 study with Africans. Breast cancers were confirmed histologically or pathologically in most studies. A classic polymerase chain reaction-restriction fragment length polymorphism (PCR-RFLP) assay was adopted in 16 of the 19 studies, however, only 37% of the included studies mentioned quality control on genotyping, such as randomly repeated assays or validation using other genotyping methods. The genotype distributions among the controls of all studies were in agreement with Hardy-Weinberg equilibrium except for two study [14], [31].

**Table 1:** Main characteristics of selected studies.

First author	Year	Country	Ethnicity	Sample size case/control	Source of controls	Genotyping method	Matching	
							Age	Sex
Sjalander	1996	Sweden	—	212/689	Population	PCR-RFLP	Yes	Yes
Weston	1997	America	European	65/117	Hospital	PCR-RFLP	Yes	Yes
Wang-Gohrke	2002	Germany	—	563/549	Population	PCR-RFLP	Yes	Yes
Suspitsin	2003	Russia	European	529/249	Hospital	PCR-RFLP	Yes	Yes
Buyru	2007	Turkey	—	115/63	Population	PCR-RFLP	Yes	Yes
Zhang	2007	China	Asian	83/167	Population	PCR-RFLP	Yes	Yes
Cavallone	2008	Canada	European	157/112	Population	Sequence	-	Yes
Costa	2008	Portugal	—	261/656	Hospital	PCR-RFLP	Yes	Yes
Gaudet	2008	America	—	578/390	Population	PCR-RFLP	Yes	Yes
De Vecchi	2008	Italy	—	350/352	Hospital	PCR-RFLP	-	Yes
Akkiprik	2009	Turkey	European	97/107	Population	AS-PCR	Yes	Yes
Hrstka	2009	Czech	—	117/108	Hospital	PCR-RFLP	Yes	Yes
Ma	2009	China	Asian	117/123	Hospital	PCR-RFLP	Yes	Yes
Bisof	2010	Croatia	—	95/108	Population	Taqman	Yes	Yes
Trifa	2010	Tunisia	African	159/132	Population	PCR-RFLP	-	Yes
Jakubowska	2010	Poland	European	311/287	Population	PCR-RFLP	Yes	Yes
Alawadi	2010	Syrians&Kuwaiti	Asian	229/133	Hospital	PCR-RFLP	Yes	Yes
Faghani	2011	Iran	Asian	145/145	Population	PCR-RFLP	Yes	Yes
Cherdyntseva	2012	Russia	European	296/196	Population	PCR-RFLP	Yes	Yes

PCR-RFLP, polymerase chain reaction-restriction fragment length polymorphism; AS-PCR, allele specific- polymerase chain reaction.

### Meta-analysis

**Figure 3:**
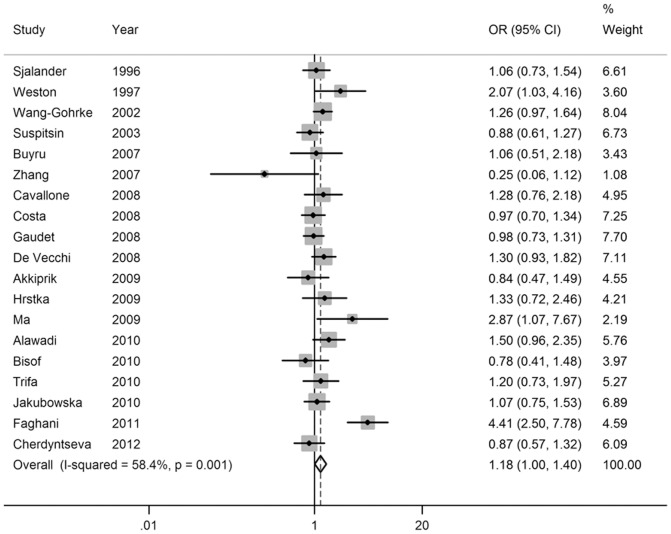
Forest plot of breast cancer risk associated with the *p53* intron 3 16-bp duplication polymorphism (Del/Ins vs Del/Del).

**Figure 4:**
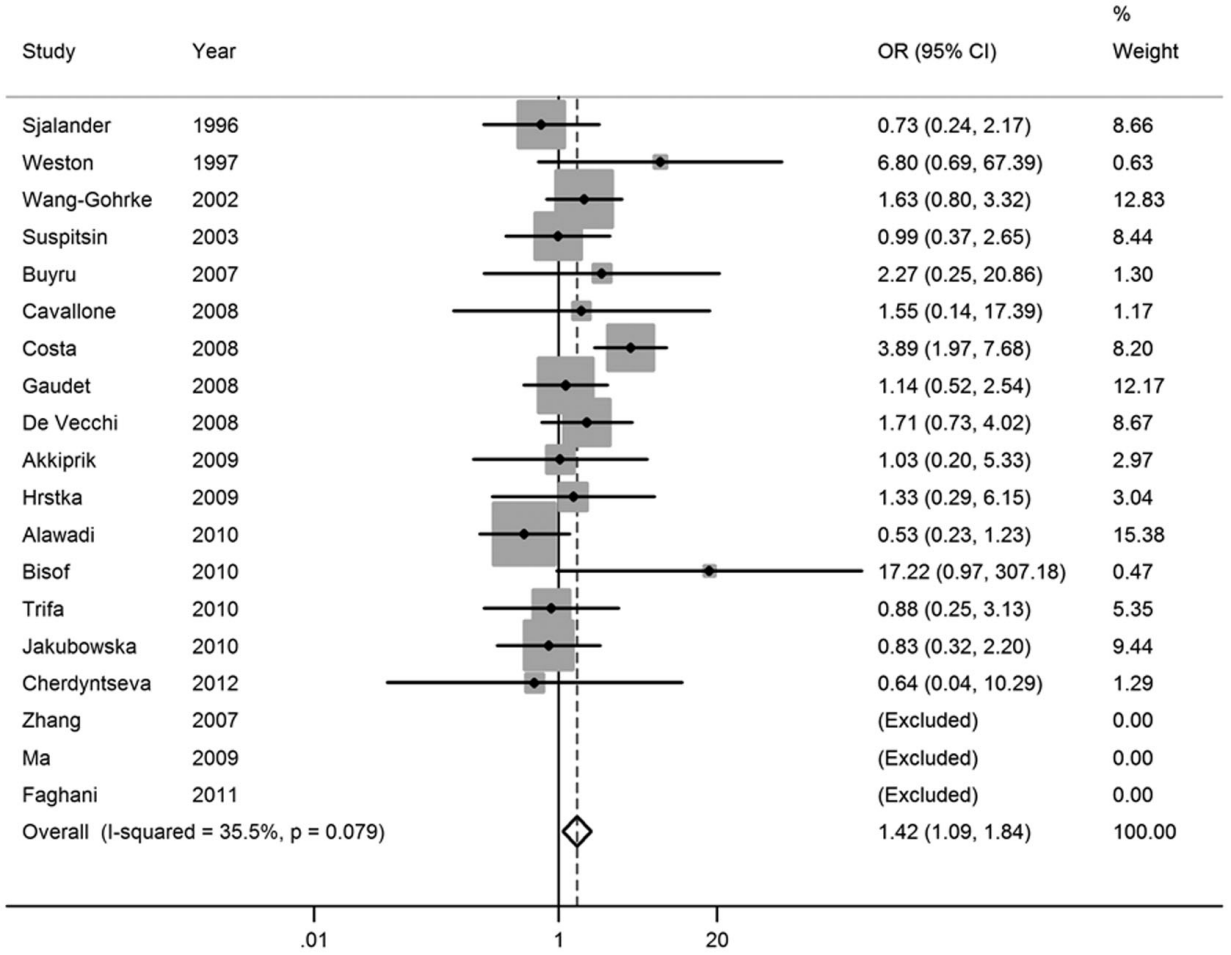
Forest plot of breast cancer risk associated with the *p53* intron 3 16-bp duplication polymorphism (Ins/Ins vs Del/Del).

**Figure 5:**
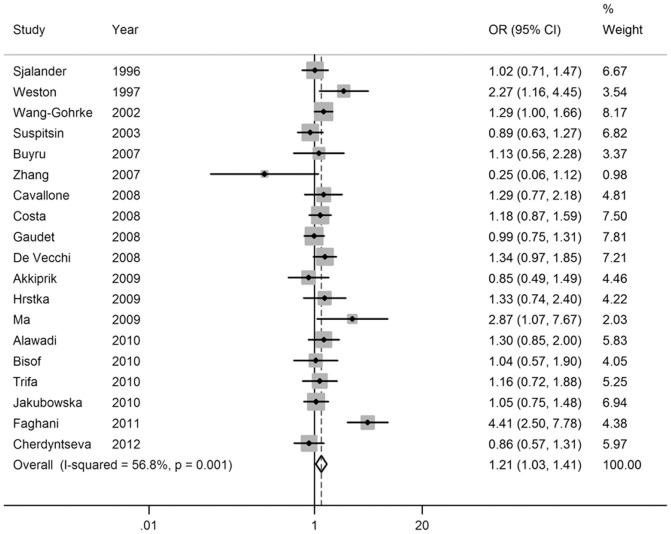
Forest plot of breast cancer risk associated with the *p53* intron 3 16-bp duplication polymorphism (Ins/Ins+Del/Ins vs Del/Del).

**Figure 6:**
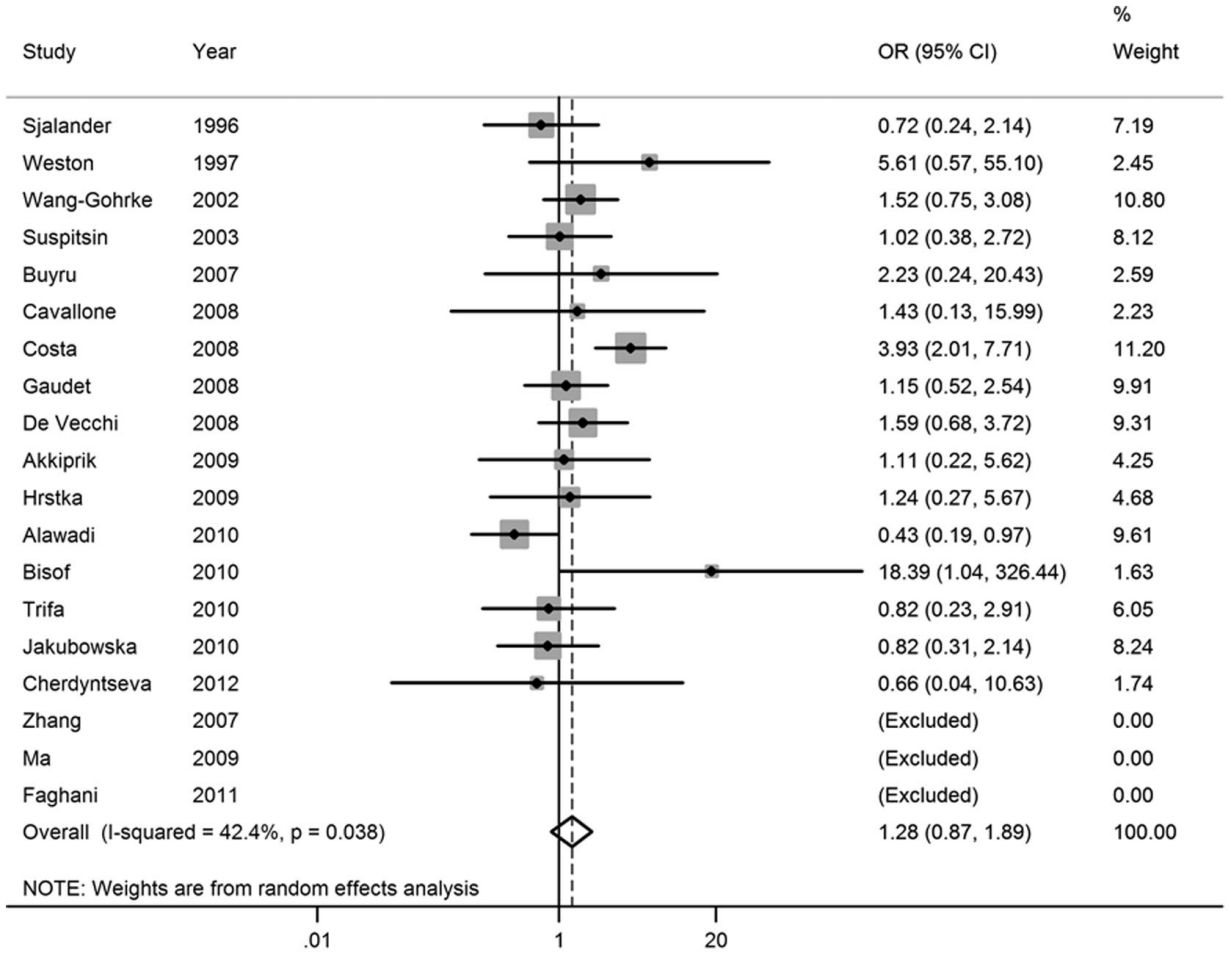
Forest plot of breast cancer risk associated with the *p53* intron 3 16-bp duplication polymorphism (Ins/Ins vs Del/Del+Del/Ins).

Pooled ORs and heterogeneity test results for the association of SNP rs17878362 and breast cancer risk were shown in Table 2. Overall, there was evidence of an association between the variant genotypes and the increased breast cancer risk in different genetic models. As shown in Table 2, the variant genotypes (Del/Ins and Ins/Ins) were associated with a significantly increased risk of breast cancer in a dose-response manner compared with the wild-type Del/Del genotype (OR = 1.18, 95% CI: 1.00–1.40 for Del/Ins and 1.42, 1.09–1.84 for Ins/Ins; *P_t_*_rend_<0.001; Figure 3 and 4). In addition, significant main effects were also observed in dominant model (OR = 1.21, 95% CI = 1.03–1.41; Figure 5), but not in recessive model (OR = 1.28, 95% CI = 0.87–1.89; Figure 6). In the stratified analysis by ethnicity, no significant associations were not observed for any genetic models (Table 2).

**Table 2:** Results of pooled ORs in the meta-analysis.

Variables	N^a^	Del/Ins vs. Del/Del		Ins/Ins vs. Del/Del		Ins/Ins+Del/Ins vs. Del/Del		Ins/Ins vs. Del/Del+Del/Ins	
		OR (95% CI)	*P* b	OR (95% CI)	*P* b	OR (95% CI)	*P* b	OR (95% CI)	*P* b
Total	19	1.18 (1.00–1.40)	0.001	1.42 (1.09–1.84)	0.079	1.21 (1.03–1.41)	0.001	1.28 (0.87–1.89)	0.038
Ethnicities									
Asian	4	1.76 (0.73–4.28)	0.001	0.53 (0.23–1.23)	-	1.67 (0.66–4.24)	<0.001	0.43 (0.19–0.97)	-
European	6	1.04 (0.84–1.28)	0.273	1.07 (0.60–1.91)	0.699	1.05 (0.84–1.32)	0.172	1.09 (0.62–1.92)	0.774
African	1	1.20 (0.73–1.97)	-	0.88 (0.25–3.13)	-	1.16 (0.72–1.88)	-	0.82 (0.23–2.91)	-
Source of controls									
Population-based	12	1.23 (0.90–1.41)	0.001	1.26 (0.88–1.81)	0.712	1.15 (0.92–1.43)	0.001	1.24 (0.86–1.77)	0.709
Hospital-based	7	1.26 (0.99–1.61)	0.087	1.58 (0.75–3.34)	0.008	1.25 (1.07–1.46)	0.141	1.47 (0.66–3.31)	0.002
Sample size									
≤500	12	1.31 (0.95–1.80)	<0.001	1.20 (0.74–1.95)	0.271	1.33 (0.98–1.81)	<0.001	1.08 (0.67–1.75)	0.180
>500	7	1.08 (0.96–1.22)	0.600	1.52 (1.11–2.08)	0.065	1.12 (0.99–1.26)	0.525	1.49 (1.10–2.03)	0.057

a Number of comparisons.

b *P* value of Q-test for heterogeneity test. Random-effects model was used when *P*-value for heterogeneity test<0.05; otherwise, fix-effects model was used.

Then, these studies were further divided into two subgroups according to their source of controls. A statistically significantly elevated risk was only found among hospital-based studies in the dominant model (OR = 1.25, 95% CI = 1.07–1.46; Table 2).

When stratifying by sample size of studies, a significantly increased risk was also observed among large sample study (>500 subjects) (homozygote model: OR = 1.52, 95% CI = 1.11–2.08; recessive model: OR = 1.49, 95% CI = 1.10–2.03) but not among small sample study (≤500 subjects).

**Figure 7:**
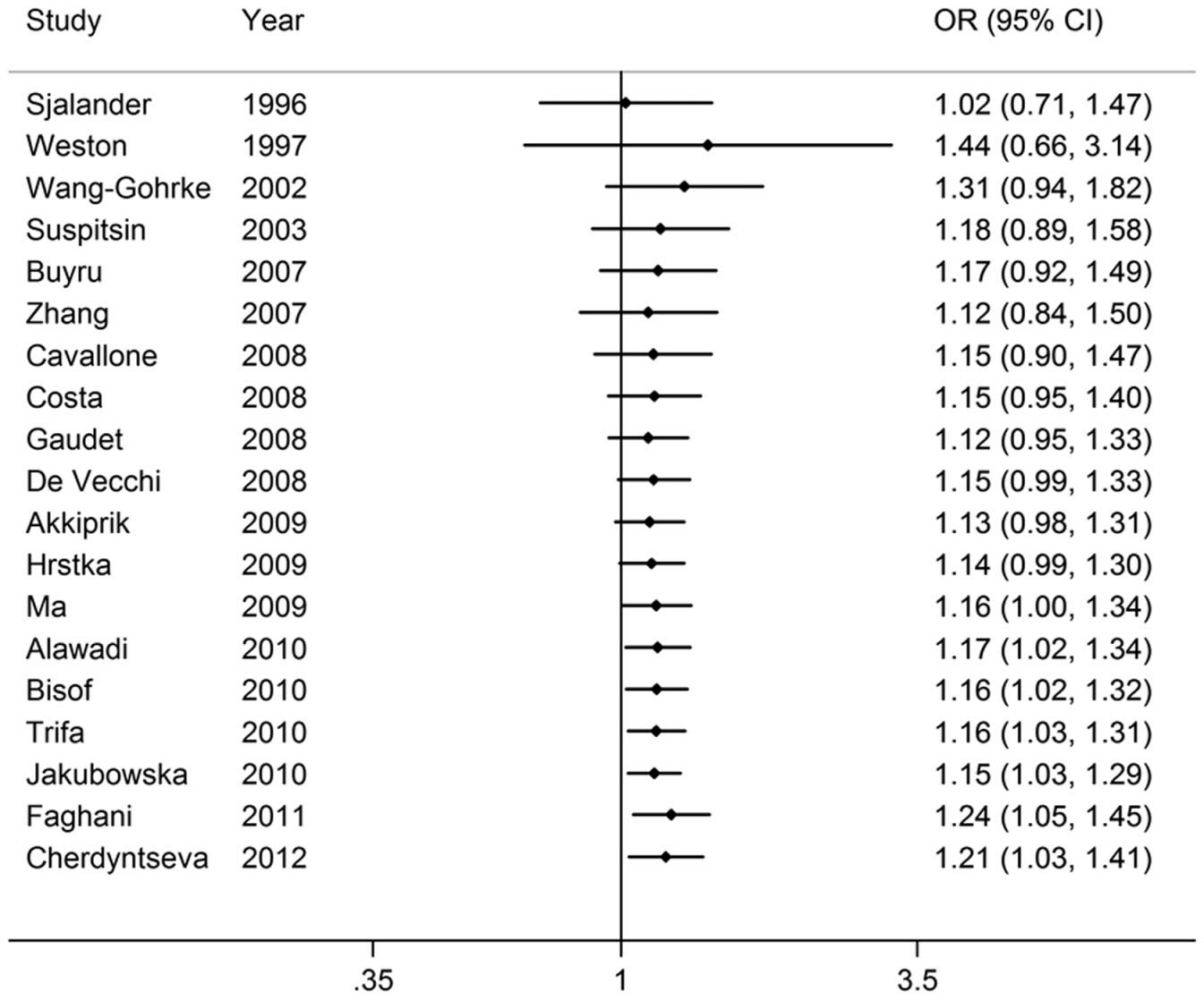
Results of the cumulative meta-analysis (Ins/Ins+Del/Ins vs Del/Del). The random effects pooled OR with 95% CI at the end of each information step is shown.

Beginning with the first published study we computed the cumulative pooled OR by stepwise addition of the results of the other available studies up to the last one published in March 2012. In the cumulative meta-analysis, the pooled OR achieved significance starting in 2009 (*P* = 0.048) and showed a trend of association as published data accumulated (*P* = 0.017, Figure 7).

### Sensitivity analysis

The leave-one-out sensitivity analysis indicated that the study by Faghani et al. [31] was the main source of heterogeneity, exclusion of which effectively abrogated the heterogeneity (Ins/Ins vs Del/Del+Del/Ins: *P* = 0.263 for heterogeneity). Although the genotype distribution in two studies [14], [31] did not follow Hardy-Weinberg equilibrium, the corresponding summary ORs were not substantially altered with or without including these studies (data not shown), indicating that the results of this meta-analysis were statistically robust.

### Publication bias

**Figure 8:**
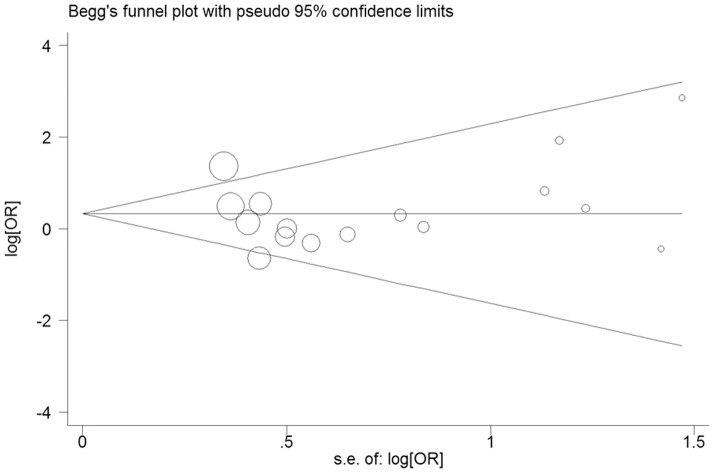
Funnel plot for publication bias test (Ins/Ins vs Del/Del).

Funnel plots and Egger's tests were conducted to estimate the publication bias of literatures. The symmetry plots indicated that there was no publication bias in all pooled studies. The results were further confirmed by Egger's test (t = 0.150, *P* = 0.882 for Ins/Ins vs Del/Del; Figure 8).

## Discussion

Our present meta-analysis incorporating 19 case-control studies (4479 cases and 4683 controls) examined the association between a commonly studied 16-bp duplication polymorphism within intron 3 of *p53* gene and breast cancer risk. The results showed that, in overall, the vaiant Ins allele was associated with a significantly increased risk of breast cancer. Given the important role of p53 in multiple cellular functions, such as DNA repair and apoptosis, it is biologically plausible that genetic variations of *p53* gene may modulate the risk of breast cancer.

It is reported that the 16-bp Ins allele was associated with lower level of *p53* transcript in lymphoblastoid cell lines, suggesting that this polymorphism causes an alteration in messenger RNA (mRNA) processing [10]. Moreover, the intron 3 16-bp duplication polymorphism was in strong linkage disequilibrium with the well-studied codon 72 variant and investigators have showed that a *P53* haplotype (codon 72 Arg/Pro, intron 6 G>A and intron 3 duplication) is associated with reduced apoptotic and DNA repair capacity in lymphoblastoid cell lines [33]. These experimental data indicate that the *P53* variants may affect P53 function. Thus, it is reasonable that the 16-bp Ins allele might result in alteration of *p53* gene expression and function, leading to decrease of p53 mediated apoptosis of tumor cells. In our present meta-analysis, we found that individuals with the 16-bp Ins allele were associated with higher risk of breast cancer than subjects with the Del allele, which was consistent with experimental findings.

Our results showed that the Ins allele may increase risk of breast cancer, which were consistent with a previous meta-analysis of eight studies based on breast cancer [34]. In the previous meta-analysis, however, the pooled sample size was relatively small and not enough information was available for more exhaustive subgroup analysis. Thereafter, several studies with a large sample size about this polymorphism on breast cancer risk have been reported, which would greatly improve the power of the meta-analysis of this polymorphism. Subgroup analyses performed by ethnicity, subject source, and sample size were also possible now. Thus, we updated this meta-analysis to derive a more precise estimation of these associations.

In our analysis, many studies did not provide the ethnic background of their participants, which precluded more detailed analysis of this polymorphism in different ethnicities. Thus, more specific ethnical information should be provided in further studies, which should lead to better understanding of the association between the 16-bp duplication polymorphism of p53 and breast cancer risk among different ethnicities.

We found an evidence for the association between the 16-bp Ins allele and breast cancer risk among large sample studies (>500 subjects) but not among small sample studies (≤500 subjects). This is probably because studies with small sample size may have limited statistical power to detect a small effect or may have generated a fluctuated risk estimate. Thus, the use of a proper and large sample study is very crucial in reducing biases in such association studies.

In this meta-analysis, a thorough sensitivity analysis was carried out by removing each single study from pooled data and the results showed that there was no influence of the individual data on overall results. Moreover, we also calculated the overall pooled ORs on 16-bp duplication with and without the two largest studies [16], [21], and in both instances, found that the 16-bp Ins allele was associated with the increased risk of breast cancer.

Some limitations of this meta-analysis should be acknowledged. First, in the subgroup analyses, the number of Africans and Asians was relatively small, not having enough statistical power to explore the real association. Second, misclassifications on disease status and genotypes may also influence the results, because cases in several studies were not confirmed by pathology, and the quality control of genotyping was also not well documented in several studies. In spite of these, our present meta-analysis also had some advantages. First, substantial number of cases and controls were pooled from different studies, which greatly increased statistical power of the analysis. Second, the quality of case-control studies included in this meta-analysis was satisfactory according to our selection criteria. Third, we did not detect any publication bias indicating that the whole pooled result should be unbiased.

Taken together, this meta-analysis provided evidence that the 16-bp duplication polymorphism within intron 3 of *p53* gene was significantly associated with an increased risk of breast cancer. Future well-designed large studies were warranted to validate these findings in different ethnic populations.
